# Detection of Ochratoxin A in Maize and Its Potential Impact on Avian Pathology in Romanian Farms

**DOI:** 10.3390/life14111477

**Published:** 2024-11-13

**Authors:** Silviu-Ionut Beia, Violeta Alexandra Ion, Elvira Gagniuc, Oana-Crina Bujor, Elena Ştefania Ivan, Andreea Barbu, Elena Pitoiu, Violeta Elena Beia, Liliana Bădulescu

**Affiliations:** 1 Faculty of Management and Rural Development, University of Agronomic Sciences and Veterinary Medicine of Bucharest, 59 Marasti Blvd., 011464 Bucharest, Romania; beia.silviu@managusamv.ro; 2 Research Center for Studies of Food Quality and Agricultural Products, University of Agronomic Sciences and Veterinary Medicine of Bucharest, 59 Marasti Blvd., 011464 Bucharest, Romania; oana.bujor@qlab.usamv.ro (O.-C.B.); elena.ivan@qlab.usamv.ro (E.Ş.I.); andreea.stan@qlab.usamv.ro (A.B.); liliana.badulescu@qlab.usamv.ro (L.B.); 3 Department of Pathology and Forensic Medicine, University of Agronomic Sciences and Veterinary Medicine of Bucharest, 59 Marasti Blvd., 011464 Bucharest, Romania; 4 Synevovet Laboratory, 81 Pache Protopopescu Blvd., 021408 Bucharest, Romania; elena.pitoiu@synevo.com; 5 National Sanitary Veterinary and Food Safety Authority, 1 Piaţa Presei Libere, 013701 Bucharest, Romania; beia.violeta@ansvsa.ro

**Keywords:** OTA, mycotoxins, method validation, maize, histopathology, ultra-performance liquid chromatography, immunoaffinity column

## Abstract

Ochratoxin A (OTA) is a nephrotoxic mycotoxin that commonly contaminates maize, posing significant health risks to both poultry and humans. In this study, a rapid and sensitive method utilizing ultra-performance liquid chromatography coupled with fluorescence detection (UPLC-FLD) was developed for the quantification of OTA levels in maize. The method utilizes immunoaffinity column purification for improved specificity. Accuracy and precision were validated in line with European Union Reference Laboratory (EURL-MP) guidelines, meeting regulatory standards for linearity, trueness, detection and quantification limits, precision, and uncertainty, as per European Commission Regulation (EC) No. 401/2006 and its amendments. The method demonstrated an average recovery rate of 116.78% for maize, with RSD_wR_ values (within-laboratory reproducibility) of 12.72%. Furthermore, OTA occurrence and its possible effects were investigated in several farms in South Romania, where necropsy and histopathological analyses of poultry revealed severe kidney damage, including renal tubular degeneration.

## 1. Introduction

Mycotoxins are natural contaminants produced as secondary metabolites by certain genera of filamentous fungi, primarily *Aspergillus*, *Penicillium* [1], and *Fusarium*. More than 200 fungal species and approximately 300 distinct fungal metabolites have been identified as mycotoxins, including aflatoxins (AFs), ochratoxin A (OTA), zearalenone (ZEA), and fumonisins [2]. These fungi are generally classified into “field fungi”, like *Fusarium*, which infect crops during growth, and “storage fungi”, such as *Aspergillus* and *Penicillium*, which contaminate grains during storage [3,4]. *Aspergillus* is a large genus with over 100 species, some of which thrive in soil or decaying vegetation, while others are responsible for plant diseases and storage contamination. Mycotoxins pose serious health risks to humans and animals due to their toxic effects [5]. OTA is classified as a possible human carcinogen (Group 2B) and has been linked to a range of toxic effects, including hepatotoxicity, teratogenicity, neurotoxicity, immunotoxicity, and enterotoxicity in various animal species [6]. The European Union (EU) has classified mycotoxins as priority substances, recognizing them as more hazardous to health than common food and feed contaminants like pesticides, preservatives, and food additives [7,8]. Common sources of exposure include cereals, nuts, dried fruits, spices, coffee [9,10,11], grapes, fish [12], wine, beer [13], mollusks, herbs, and feed materials [14]. Given the significant impact of mycotoxins, particularly OTA, on agricultural products, it is crucial to explore these toxins in greater detail due to their serious implications for food safety and animal health. The fungi that produce OTA can thrive in temperatures ranging from 0 to 37 °C [13], and OTA is highly thermally stable, making its removal from feed challenging even at temperatures up to 250 °C [15]. Storage conditions play a critical role in preventing OTA contamination in agricultural products. High humidity and extended storage periods encourage fungal growth and OTA production, while low humidity and adequate ventilation reduce these risks. Effective storage practices include using airtight, moisture-proof containers, maintaining clean and mold-free environments, and thoroughly drying crops before storage. However, many small local farmers remain unaware of these practices, and without proper aeration their bulk storage methods often result in fungal contamination by spring.

Numerous analytical methods have been developed to investigate OTA, including capillary electrophoresis (CE) [16], gas chromatography-mass spectrometry (GC-MS) [17,18], and enzyme-linked immunosorbent assay (ELISA) [19]. However, most of the proposed methods rely on liquid chromatography (LC) coupled with various detection systems, such as fluorescence detection (LC-FLD) [20], tandem mass spectrometry (LC-MS/MS) [21], and high-resolution mass spectrometry (LC-HRMS) [22].

Sample extraction for OTA typically involves liquid–liquid extraction (LLE) or solid–liquid extraction (SLE), followed by cleanup with immunoaffinity columns (IAC) or solid-phase extraction (SPE) cartridges [22]. For OTA determination, traditional methods involve mixing a ground sample with an acetonitrile–water mixture, filtering, and applying the extract to a purification column [17]. SPE effectively isolates mycotoxins using sorbents such as C8, C18, aluminum oxide, and silica gel. Advanced SPE techniques, including magnetic SPE (m-SPE) and dispersive SPE (d-SPE), have been utilized to detect numerous mycotoxins and pesticides [21,23]. Additionally, the QuEChERS (Quick, Easy, Cheap, Effective, Rugged, and Safe) method simplifies mycotoxin detection by combining extraction, separation, and purification in one run, offering high throughput and significant recovery [21]. Understanding the extent and implications of OTA contamination relies heavily on the effectiveness of the analytical methods used to detect and measure this mycotoxin. The accurate detection of OTA in food and feed is crucial due to its significant health risks. Despite advances in analytical techniques, the primary concern remains the potential impact of OTA on animal and human health.

Feed ingestion is the primary route of mycotoxin exposure in animals [24]. Various mycotoxins, including aflatoxin B1 (AFB1), aflatoxin M1 (AFM1), citrinin (CIT), dihydrocitrinone (DH-CIT), deoxynivalenol (DON) conjugates, enniatin B (ENNB), and OTA, have been detected in animal biological fluids [25,26]. This presence suggests that animals and humans may also be exposed to mycotoxins through skin contact or inhalation of aerosols [27].

Poultry exhibit a lower absorption rate of OTA but are more sensitive to its effects compared to mammals [5]. OTA exerts significant nephrotoxic effects on broilers, leading to kidney enlargement and hyperplasia of the tubular epithelium [28,29]. It also adversely affects the immune system of birds, causing leukocytopenia [5,30]. The effect of OTA on growth includes a decreased body weight, poor feed conversion ratio, stunted growth, low-quality eggshells, reduced egg production, and immunosuppression. Furthermore, it can cause liver damage and affects gastrointestinal health and microbial communities [30,31]. Clinical signs of OTA toxicity in poultry include weakness, anemia, decreased feed consumption, reduced growth rates, poor feathering, and elevated mortality rates at high dietary concentrations [32,33]. Pathophysiological changes associated with OTA exposure include decreased urine concentration, a reduced glomerular filtration rate, impaired proximal tubular function, and renal degeneration with ultrastructural alterations. Additionally, studies have reported increases in the relative weights of the liver, spleen, pancreas, proventriculus, gizzard, and testes in poultry fed OTA [34].

The aim of this study was to develop and implement a rapid and reliable method for the qualitative and quantitative detection of mycotoxins in maize used in intensive farming systems. Access to rapid diagnostic methods enables farmers to obtain timely toxicological results for maize, especially in cases where mycotoxicosis is suspected, allowing for swift intervention to minimize economic losses. Currently, in Romania, mycotoxicological analysis of maize requires a lengthy processing time, with an average confirmation period of approximately 21 days. The method developed in this study significantly reduces this timeframe to just 2 days, offering a faster and more efficient approach to OTA detection. The UPLC-FLD method was validated in line with European Union Reference Laboratory (EURL-MP) [35] guidelines, ensuring accuracy and regulatory compliance. This study hypothesized a correlation between OTA levels in maize and poultry health issues, such as increased mortality and specific pathological changes, as observed in Romanian farms where chickens consumed mold-contaminated maize. Histopathological examinations of tissue samples from recently deceased broiler chickens revealed potential OTA-related organ damage, particularly in the kidneys, along with reduced lymphocyte populations in lymphoid organs, suggesting compromised immune responses due to chronic OTA exposure.

## 2. Materials and Methods

### 2.1. Chemicals and Reagents

The standard solution was prepared using an OTA reference material of 100 µg/mL concentration in methanol purchased from LGC Limited Standards (Middlesex, TW11 0LY, London, UK). Methanol and acetonitrile (HPLC grade) were purchased from Honeywell (Riedel-de Haën, Seelze, Germany); glacial acetic acid was purchased from Fisher Scientific (Pittsburgh, PA, USA). 

Potassium chloride was purchased from Scharlab S.L. (Sentmenat, Barcelona, Spain); sodium chloride, potassium dihydrogen phosphate, and disodium hydrogen phosphate were purchased from Carl Roth GmBH (Karlsruhe, Germany). OTAClean for Ochratoxin A immunoaffinity columns were obtained from LCTech GmbH (Obertaufkirchen, Germany). Phosphate-buffered saline (PBS, pH 7.2) was prepared by adding potassium chloride (0.20 g), potassium dihydrogen phosphate (0.20 g), anhydrous disodium hydrogen phosphate (1.16 g), and sodium chloride (8.00 g) to 900 mL of water. The pH was adjusted with 0.1 M NaOH and 0.1 M HCl. The volume was made up to 1 L with water. Tissue processing reagents included buffered 10% formaldehyde (Laurypath for Epredia, 69630 Chaponost, France), absolute ethyl alcohol, 70% and 95% ethyl alcohol, xylene (Laurypath for Epredia, 69630 Chaponost, France), paraffin wax (Histoplast LP, Epredia, Portsmouth, NH, USA), and hematoxylin and eosin (ST Infinity HE Staining kit, Leica Biosystems, Richmond, IL, USA).

### 2.2. Sample Collection

Several farms in Nuci and Moara Domnească (Ilfov County), Coșereni and Fierbinți (Ialomița County), and Lunca (Buzău County) were investigated for OTA contamination in maize used for poultry feeding (Table 1). These investigations were prompted by farmers’ reports of unusually high poultry mortality rates, leading them to submit maize samples for OTA analysis. Samples from maize stored in bulk were collected in accordance with Commission Regulation (EU) No. 2782/2023, which amends the requirements of Regulation (EC) No. 401/2006 [36], to ensure that each sample represented its respective batch.

A total of forty incremental samples were collected from various points (upper, middle, and lower areas) to capture the entire mass of stored maize. These incremental samples were then combined to form an aggregate sample of approximately 4 kg. From the aggregate sample, two subsamples of at least 0.5 kg each were taken for examination and transported under optimal conditions to the research center’s laboratory for mycotoxin analysis. Next, the samples were milled and sieved (0.5 mm) to produce representative test samples.

Among the investigated farms, the one with the highest OTA contamination, which was also selected for histopathological examination, was located in Nuci, Ilfov County. This farm housed 27,000 Ross 308 broiler chickens in a 1000 m^2^ facility. According to the farmer, the feed was switched during the second week to a maize-based diet specifically formulated for the chickens’ growth phase, which contained 60% maize.

The chickens were fed ad libitum, with continuous access to food and water. By the third week, the farm’s mortality rate had reached 3%. However, shortly after the introduction of the new feed, mortality rates spiked, rising to 10–15% by the fourth week. The chickens also exhibited a marked decline in weight gain and deterioration in overall health. Clinical signs such as lethargy, anorexia, diarrhea, refusal to feed, dehydration, and damaged plumage became increasingly apparent. To determine the cause of the increased mortality, necropsies were conducted on 10 recently deceased broiler chickens. Samples from the kidneys, liver, thymus, bursa of Fabricius, and spleen were collected from 32-day-old Ross 308 chickens for histopathological examination.

### 2.3. OTA Extraction and Clean-Up

The extraction and cleanup of OTA in maize were performed using a modified procedure based on the OTAClean immunoaffinity column from LCTech GmbH, Obertaufkirchen, Germany [37]. In brief, 2 g of NaCl and 100 mL of an 80:20 (*v*/*v*) methanol solution were added to 20 g of a ground maize sample. The mixture was stirred for 1 h and then filtered. A 12 mL portion of the filtrate was diluted with 48 mL of PBS (pH 7.4) before passing through the immunoaffinity column, which contains immobilized monoclonal antibodies specific to OTA and offers a recovery rate exceeding 90% for the OTA standard, as stated in the certificate of analysis. After the extract fully passed through the column, it was washed with 10 mL of water, and the OTA was eluted using 1.5 mL of methanol. The collected eluate was refrigerated for subsequent analysis.

### 2.4. UPLC-FLD Instrument and Chromatographic Analysis

A Waters Acquity I chromatographic system (Milford, MA, USA) was used for OTA analysis, equipped with a UPLC binary pump, autosampler, injection system, and fluorescence detector. The analysis was performed under isocratic conditions, with a total runtime of 3 min. For OTA separation, a Zorbax Eclipse Plus C18 column (100 mm × 2.1 mm, 1.8 µm) (Agilent Technologies, Santa Clara, CA, USA) was employed. The mobile phase consisted of acetonitrile/water/acetic acid (70:30:1, *v*/*v*/*v*), with a flow rate of 0.5 mL/min and an injection volume of 5 µL. Detection was carried out at an excitation wavelength of 330 nm and an emission wavelength of 460 nm. During the analysis, the samples were maintained at 20 °C, and the column temperature was set to 30 °C.

### 2.5. OTA Method Validation

The method validation process evaluated several critical parameters, including the linearity, limit of quantification (LOQ), repeatability relative standard deviation (RSD_r_), within-laboratory reproducibility relative standard deviation (RSD_wR_), and recovery (R), in compliance with Commission Regulation (EU) No. 2782/2023, which amends the requirements of Regulation (EC) No. 401/2006 [36]. Performance criteria were also benchmarked against the proposed guidelines from the draft version of the EURL-MP [35].

To assess linearity, seven concentrations of OTA standard solutions (ranging from 0.5 to 10 ng/mL in methanol: acetic acid, 98:2, *v*/*v*) were used. Regression analysis was conducted to explore the relationship between OTA concentration (independent variable) and fluorescence detector intensity (dependent variable). Data analysis was performed using Microsoft Excel (version 2013), utilizing the Analysis ToolPak’s Regression tool. Key statistical outputs included the correlation coefficient (Multiple R), the coefficient of determination (R^2^), and the adjusted R^2^. The ANOVA table was used to test the overall significance of the model, while the coefficient table provided detailed insights into variable relationships. Residual analysis was performed to confirm model fit and validate regression assumptions, with all statistical tests conducted at a significance level of α = 0.05.

According to the EURL-MP guidelines [35], the limit of quantification (LOQ) was established at levels lower than 0.5 times the maximum level (ML). Method validation was conducted on maize flour quality control material (QC) sourced from Fera Science Ltd. (Sand Hutton, York, UK), which had an assigned ochratoxin A (OTA) value of 0.810 µg/kg. This assigned value served as the LOQ for the analysis, indicating that concentrations below this threshold may not be reliably quantified. The acceptable |z|-score range for this QC material was 0.454–1.166 µg/kg. Quality control procedures for the maize samples were performed through extraction and cleanup in accordance with the developed method.

The estimation of uncertainty followed the guidelines outlined in the Eurachem Guide for Quantifying Analytical Measurement Uncertainty [38], utilizing validation data from two fortification levels. Key sources contributing significantly to the overall uncertainty—such as the standard uncertainty from control solutions, uncertainty from linearity (coefficient of variation), precision of repeatability of control solutions, bias-related uncertainty, and precision of sample reproducibility—were prioritized in calculating the combined uncertainty. 

### 2.6. Tissue Preparation and Histopathological Examination

The tissue and organ samples from 10 broiler chickens that had recently died (including the kidney, liver, thymus, bursa of Fabricius, and spleen) were fixed in buffered 10% formaldehyde for 24 h, followed by dehydration in (1) 70% ethylic alcohol for 60 min, (2) 95% ethylic alcohol for 45 min, and (3) absolute ethylic alcohol for 2 h. The clearing phase of the samples was made by repeated xylene immersions, followed by paraffin wax infiltrations [39]. The samples were automatically processed with the tissue processor Gemini AS Epredia (Runcorn, UK) and paraffin embedding was carried out with modular tissue-embedding center Microm EC 350-1 (Thermo Fischer Scientific, Waltham, MA, USA). The resulting blocks were cut at 3 μm using the RM 125RTS (Leica, Wetzlar, Hesse, Germany) microtome. The sections were stained with hematoxylin and eosin (HE) by using the Gemini AS Epredia Slide Stainer (Runcorn, UK). The examination was performed with an Olympus BX43 microscope coupled to an Olympus DP73 video camera and using the Olympus Cell^B analysis system version 3.4 (Shinjuku, Japan) for the microscopic evaluation of the samples.

## 3. Results

### 3.1. OTA Method Validation Parameters and Sample Contamination

**Figure 1:**
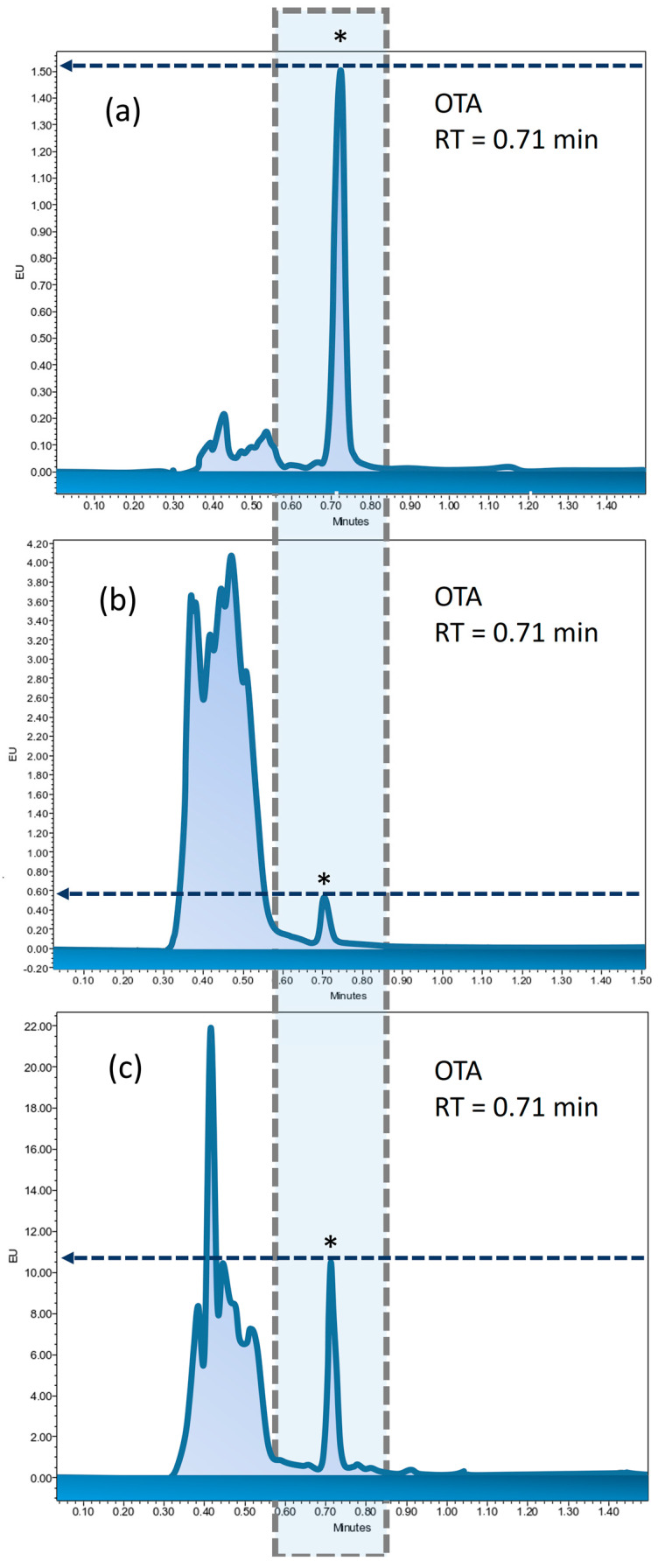
Typical UPLC-FLD chromatograms of OTA: (**a**) reference standard solution (3 ng/mL), (**b**) QC (maize), and (**c**) maize sample (M3). * represents the OTA peak.

The validation results of the OTA analytical method demonstrate that the developed approach effectively separates OTA from matrix interferences, achieving high selectivity primarily due to immunoaffinity columns that significantly reduce matrix effects. The UPLC-FLD chromatograms for the OTA reference standard solutions (3 ng/mL) demonstrate excellent resolution and similar clarity, as observed in the chromatograms for quality control (QC) maize materials and samples (Figure 1).

Under optimal conditions, the calibration curve was established using five concentration levels of the OTA reference material solution, with two replicates at each level. The linearity was confirmed within the range of 0.5–10 ng/mL, with a regression equation of y = 7436.2x + 152.69 and a correlation coefficient (R^2^) of 0.999, as per ISO 8466-1:2021 standards [40]. ANOVA analysis determined a standard error of 440.40 and a method standard deviation of 0.06, resulting in a variation coefficient of 1.37%, well below the generally accepted threshold of 5%. To evaluate accuracy, a 3 ng/mL solution from a different batch of certified OTA reference material was prepared and injected over 10 consecutive days, with two repetitions per injection. The accuracy of the method was clearly demonstrated, with results ranging from 95.83% to 104.65%.

LOQ is a crucial parameter, especially when evaluating contaminants with defined maximum levels. In this study, the LOQ was experimentally determined using trueness and precision criteria, based on maize QC material. To assess repeatability (RSD_r_), six QC samples were analyzed in a single day, resulting in an average concentration of 0.95 µg/kg and an average recovery rate of 116.78%. The RSD_r_ was calculated at 7.95%, indicating excellent repeatability at low OTA concentrations. The within-laboratory reproducibility (RSD_wR_) for the LOQ was 12.72%, which meets the requirements set by Commission Regulation (EU) No. 2782/2023, amending Regulation (EC) No. 401/2006 [36]. This falls well within the accepted RSD_r_ threshold of ≤20%, as well as the criteria specified in the EURL-MP guidelines [35], which recommend an LOQ value lower than 50 µg/kg.

From the nine maize samples tested, three samples were below the limit of quantification (LOQ)—two from Lunca, Buzău County, and one from Fierbinți. Additionally, three samples had OTA concentrations lower than 5 µg/kg: one from Moara Domnească at 4.88 µg/kg, and two from Nuci, Ilfov County, with levels of 1.47 µg/kg and 1.77 µg/kg, respectively. The sample collected from Coșereni contained 64.60 µg/kg of OTA, while another sample from Fierbinți showed a concentration of 14.68 µg/kg.

Notably, one maize sample (M3) from Nuci, Ilfov County, recorded a concentration of 228.75 µg/kg (Table 2), which is close to the maximum recommended OTA limit of 0.25 mg/kg established by Commission Recommendation No. 2016/1319 [41]. Consequently, poultry samples were collected from this farm to look into the possible impact of OTA-contaminated feed on kidney health. The OTA concentration in the feed mix was calculated using the fact that maize accounted for 60% of the diet specifically formulated for the chickens’ growth phase. Among the nine samples tested, sample M3 exhibited a concerning concentration of 137.25 µg/kg, while sample M7 contained 38.76 µg/kg, warranting significant attention. Furthermore, sample M9 showed a moderate contamination level of 8.81 µg/kg.

### 3.2. Gross and Histological Sample Result

**Figure 2:**
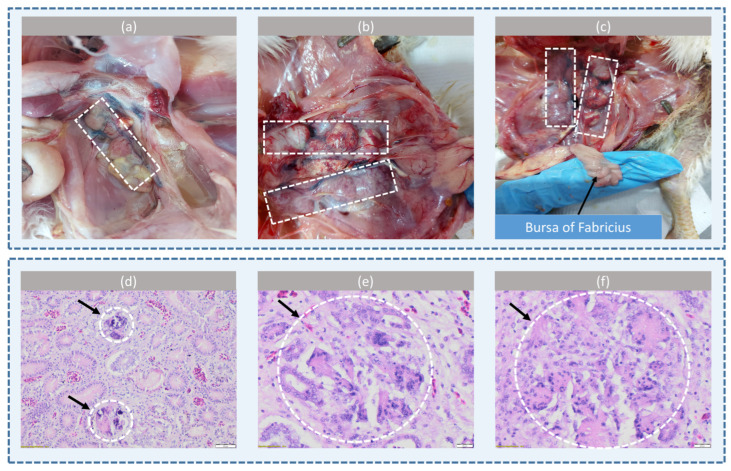
Gross and histopathological lesions from Ross 308 gout-affected commercial broiler chickens after exposure to feed with mycotoxins. (**a**) Swollen kidneys full of chalky deposit and the dilatation of ureters; the kidneys are marked by dashed white-line box. (**b**) The kidneys are enlarged, degenerate, and filled with urates, with bilateral multifocal petechiae; the kidneys are marked by the dashed white-line box. (**c**) The gross examination of the bursa of Fabricius reveals significant atrophy, with a notable reduction in size and weight, and pale discoloration; the kidneys are marked by the dashed white-line box. (**d**) Visceral gout, observed in the transverse cross-section of the chicken kidney. Bluish-purple urate deposits in the lumen of renal tubules forming urate cylinders (arrows), HE stain. (**e**) Gout tophi (indicated by a white dashed circle and arrow), with cellular spicules surrounded by macrophages and multinucleated giant cells, moderate interstitial lymphocytic and histiocytic nephritis, tubular degeneration, and necrosis in the chicken kidney, HE stain; (**f**) Gout tophi (indicated by a white dashed circle and arrow), with interstitial fibrosis and urate deposits in the chicken kidney, HE stain.

During the necropsy of chickens from the farm located in Nuci, Ilfov County, where elevated levels of OTA (228.75 µg/kg) were detected in the contaminated maize, several pathological changes were observed. The kidneys of all individuals showed lesions marked by enlargement, degeneration, pallor, and increased firmness, with chalky deposits accumulating in the ureters (Figure 2). The serous membranes exhibited chalky white, fine, dry deposits. The liver showed hepatomegaly with a yellow-green coloration, characteristic of hepatic steatosis. Numerous foci of necrosis were seen on the surface of the liver, and on section, the liver tissue was soft and friable, suggesting massive degeneration of hepatocytes. Lymphoid organs such as the spleen, bursa of Fabricius, and thymus were reduced in size and pale.

Microscopic examination revealed multifocal and random deposits in the kidneys, including colorless to intensely basophilic, spheroid, or sharp acicular crystalline deposits (urate tophi). Surrounding these deposits there were a moderate number of inflammatory cells, predominantly mononuclear lymphocytes, active macrophages, multinucleated giant cells, and heterophils mixed with eosinophilic and karyolytic cellular debris. Extravasated red blood cells extended into the adjacent interstitium. Renal tubules exhibited mild to moderate ectasia with a degenerate epithelium, enlarged epithelial cells with indistinct borders and microvacuolated cytoplasm, hypereosinophilic epithelial cytoplasm, and pyknotic nuclei. The interstitium contained infiltrates of lymphocytes and macrophages, along with signs of tissue fibrosis.

In the liver, hepatocytes displayed moderately increased cell volume with finely granular cytoplasm, indicative of extralobular hepatic glycogenosis. Some hepatocytes contained microvacuoles or macrovacuoles (steatosis), accompanied by perivascular, portobiliary, and sinusoidal inflammatory infiltrates. Multifocal hepatocytic necrosis was also noted. The intestinal mucosa was swollen and exhibited multiple hemorrhagic spots. In certain segments, the mucosa was necrotic and covered by a fibrinous exudate. Occasionally, the glandular body (proventriculus) showed erosions and ulcerations. A reduction in lymphocyte populations was observed in the lymphoid organs. The bursa of Fabricius frequently showed hypocellular lymphoid follicles with moderate to severe lymphocyte depletion and signs of cellular apoptosis. 

## 4. Discussion

The UPLC-FLD technique serves as an affordable and user-friendly alternative to mass spectrometry (MS) or tandem mass spectrometry (MS/MS), while still delivering high specificity and sensitivity. This makes it particularly effective for detecting mycotoxins in various matrices, including maize, and suitable for multi-mycotoxin analyses. Recently, multiresidue immunoaffinity columns (IACs) have been developed to streamline the simultaneous purification of multiple mycotoxins, including aflatoxins B1, B2, G1, G2, OTA, and zearalenone [42]. Although these columns improve workflow efficiency, their recovery rates tend to be lower compared to single-target columns. OTA-specific immunoaffinity columns, however, remain highly effective for analyzing complex feed matrices, offering superior selectivity and enhanced analytical reliability. In addition, alternative methods such as QuEChERS combined with solid-phase extraction (SPE) have been explored as substitutes for IAC; however, they do not match the overall effectiveness. Ultimately, despite the higher cost associated with IAC, it continues to be regarded as the most effective and environmentally friendly option for mycotoxin analysis [43].

In our study, the UPLC-FLD method was validated for precision and accuracy, effectively separating OTA from matrix interferences while demonstrating high selectivity through the use of immunoaffinity columns. The method also enabled rapid analysis, with OTA showing a retention time of just 0.71 min and a total instrumental analysis time of 3 min. The UPLC chromatograms exhibited excellent resolution, high linearity (R^2^ = 0.999), and accuracy ranging from 95.83% to 104.65%.

Leite et al. (2023) [44] developed and optimized a sensitive, precise, and robust multi-mycotoxin analytical method using ultra-high-performance liquid chromatography coupled with tandem mass spectrometry (UHPLC-MS/MS) for the detection of 23 regulated, non-regulated, and emerging mycotoxins in maize grain, including ochratoxin A (OTA). This approach used QuEChERS extraction with a C18 sorbent, followed by UHPLC-MS/MS analysis to accurately quantify these mycotoxins in maize samples. By analyzing blank samples and using a signal-to-noise ratio of 10:1, Leite et al. (2023) [44] achieved a limit of quantification (LOQ) of 3.7 µg/kg, with recovery rates between 75.8% and 98.1%. In comparison, our study achieved a significantly lower LOQ of 0.81 µg/kg, along with a recovery rate of 116.78%.

Another key validation parameter, RSD_wR_, which influences the standard uncertainty of the method, was assessed in this study. In our research, we observed an RSD_wR_ of 12.72% at 0.81 µg/kg, while Silva et al. (2019) [45] reported a slightly higher RSD_wR_ of 14.5% at a concentration of 1.5 µg/kg. Leite et al. (2023) [44] recorded an RSD_wR_ of 13.6% in a poultry feed analysis spiked at 2 µg/kg, further underscoring the influence of feed matrices on analyte recovery rates.

Romania’s warm and humid climate, especially in regions such as the Southern Plain and Dobrogea, creates optimal conditions for *Aspergillus* species, key OTA producers. These conditions increase the risk of OTA contamination, particularly during maize storage in silos, posing potential health risks if not effectively managed. Between January 2012 and July 2015, post-harvest OTA contamination in Romanian maize was reported at an incidence rate of 6.8%, with 4 out of 59 samples testing positive (Gagiu et al., 2018) [46]. OTA levels in contaminated samples ranged from below 2.50 µg/kg to a maximum of 6.72 µg/kg, with a mean of 2.70 ± 0.43 µg/kg and a median below 2.50 µg/kg. Of the samples, 6.8% exceeded the maximum allowable OTA level, raising food safety concerns in affected areas. Processed maize products showed the highest OTA levels, with maize grains stored in silos reaching up to 3.39 µg/kg in 2012 and corn germ samples reaching 5.64–6.72 µg/kg in 2014.

Maize, however, is not the only feed susceptible to OTA contamination. Alexa et al. (2013) [47] found a relatively low OTA incidence in 2010 wheat samples in western Romania, with a maximum contamination level of 11.3 µg/kg. However, OTA incidence increased significantly in 2011, with 92.31% of wheat samples testing positive and contamination levels reaching up to 25.70 µg/kg, demonstrating annual fluctuations in contamination across feed types. OTA contamination has also been reported in maize from other regions, indicating similar risks in various climates. For instance, Kortei et al. (2021) [48] assessed OTA levels in Ghanaian maize, collecting samples from village markets from July to December 2020. Of the 180 samples analyzed, 103 contained OTA, with contamination levels ranging from 0 to 97.51 µg/kg. Of these, 52.22% exceeded the EFSA tolerable limit, while 49.44% exceeded Ghana’s safety standard.

In Doha, Qatar, Alkuwari et al. (2021) [49] reported that all maize samples (n = 13) collected from local markets tested positive for OTA, with levels between 1.55 and 2.58 µg/kg. Similarly, Beg et al. (2006) [50] investigated OTA contamination in yellow maize and other feed ingredients in a Kuwaiti poultry feed production facility. Among the 14 broiler starter feed samples, 93% contained less than 5 µg/kg OTA, and 32 maize samples analyzed showed that half had OTA concentrations below this threshold.

The nephrotoxic effects of ochratoxins have been investigated for a long time. Grossly, these lesions include enlarged kidneys with urate retention. Microscopically, renal tubular degeneration and necrosis, along with cast formation and urate tophi, are typically observed. Lymphoid organ suppression (lymphocyte depletion) and hepatic necrosis may also occur. Similar results have been reported by other studies [51]. Santin et al. (2002) [52] conducted a necropsy analysis of chickens fed a diet containing OTA at 2 mg/kg for three to four weeks, observing alterations in internal organs such as pale, swollen kidneys and enlarged, yellowish, friable livers. Histopathological examinations revealed the vacuolation and megalocytosis of hepatocytes, hyperplasia of the biliary epithelium, and hypertrophy of renal proximal tubular epithelial cells. In another study, broiler chickens fed a diet with 2 mg/kg of OTA showed no changes within the first three weeks of treatment [53]. However, significant changes were noted after five weeks, including kidney swelling, slight liver enlargement, and a reduction in the size of the bursa of Fabricius. Microscopic analyses indicated marked changes in lesion scores, with the highest scores observed in the kidneys, followed by the liver, bursa, spleen, and thymus. Kumar et al. (2004) [54] confirmed that OTA is more nephrotoxic than hepatotoxic for broilers, with the most significant effects seen in animals fed a diet contaminated with OTA at 2 mg/kg. These effects included the atrophy of the bursa, thymus, and spleen, along with lymphocyte depletion. In a recent study on two commercial poultry farms, Bozzo et al. (2008) [55] detected OTA in all feed samples, with concentrations ranging from 0.160 to 0.332 mg/kg. The OTA-contaminated feed was administered to the animals for at least two months. Postmortem inspections, along with cytological and histological examinations of layer hens, evidenced gross and microscopic lesions in the kidneys and liver. Sawale et al. (2009) [56] reported negative effects of OTA on performance, hematobiochemical disturbances, and severe immunosuppression in laying hens fed a diet contaminated with OTA at 1 mg/kg for 60 days. Our study provides additional evidence in this field.

## 5. Conclusions

This study presents a developed and validated UPLC-FLD method that exhibits robust precision and accuracy in the detection of OTA in maize. The method effectively separates OTA from matrix interferences, thereby ensuring reliable detection at low concentrations. The maize samples tested from various counties in Romania reveal significant variability in OTA concentrations. Notably, maize sample M3 exhibited a concentration of 228.75 µg/kg, which, while below the maximum recommended limit of 250 µg/kg, underscores the potential risk associated with OTA contamination in maize. Histopathological assessments of the collected poultry samples indicated nephrological damage that may be associated with OTA presence, including kidney enlargement, urate deposits, and tubular degeneration, along with lymphoid organ suppression and hepatic necrosis. These findings corroborate existing research on OTA’s severe impact on poultry health. This study underscores the necessity for stringent feed testing and management practices to address OTA contamination and mitigate its adverse effects. Furthermore, the analysis highlights the effectiveness of the UPLC-FLD method in providing the accurate quantification of mycotoxins, with high correlation coefficients and recovery rates affirming its reliability. This research emphasizes the critical importance of continuous mycotoxin surveillance to ensure feed safety and protect both animal and human health, offering valuable insights for the agricultural and public health sectors.

## Figures and Tables

**Table 1:** Samples used for OTA analysis.

Sample Code	Variety	Storage Conditions	Location
M1	Carioca	Bulk in storage	Moara Domnească/ Ilfov
M2	P9889	Classic horizontal hall	Nuci/Ilfov
M3	P9889	Classic horizontal hall	Nuci/Ilfov
M4	P9889	Classic horizontal hall	Nuci/Ilfov
M5	P9889	Vertical silo made of galvanized sheet metal	Lunca/Buzău
M6	P9889	Horizontal silo made of concrete and masonry, warehouse type	Lunca/Buzău
M7	P9889	Bulk in storage	Coşereni/Ialomiţa
M8	P9889	Bulk in storage	Fierbinţi/Ialomiţa
M9	P9889	Bulk in storage	Fierbinţi/Ialomiţa

**Table 2:** Ochratoxin A (OTA) contamination levels in maize samples from selected farms.

Sample Code	OTA in Maize * (µg/kg)	OTA in Feed Mix ** (µg/kg)
M1	4.88	2.93
M2	1.77	1.06
M3	228.75	137.25
M4	1.47	0.88
M5	<LOQ	-
M6	<LOQ	-
M7	64.60	38.76
M8	<LOQ	-
M9	14.68	8.81

Legend: <LOQ—below limit of quantification (0.810 µg/kg). * OTA in maize was analytically determined using UPLC-FLD. ** OTA in feed mix was calculated based on percentage of maize in maize-based diet formulated specifically for chickens’ growth phase.

## Data Availability

Data are available on request from the authors.
